# Statistical Multipath Model Based on Experimental GNSS Data in Static Urban Canyon Environment

**DOI:** 10.3390/s18041149

**Published:** 2018-04-10

**Authors:** Yuze Wang, Xin Chen, Peilin Liu

**Affiliations:** Shanghai Key Laboratory of Navigation and Location Based Service, School of Electronic Information and Electrical Engineering, Shanghai Jiao Tong University, Shanghai 200240, China; wangyuze90@sjtu.edu.cn (Y.W.); liupeilin@sjtu.edu.cn (P.L.)

**Keywords:** global navigation satellite system, multipath, statistical model, urban canyon

## Abstract

A deep understanding of multipath characteristics is essential to design signal simulators and receivers in global navigation satellite system applications. As a new constellation is deployed and more applications occur in the urban environment, the statistical multipath models of navigation signal need further study. In this paper, we present statistical distribution models of multipath time delay, multipath power attenuation, and multipath fading frequency based on the experimental data in the urban canyon environment. The raw data of multipath characteristics are obtained by processing real navigation signal to study the statistical distribution. By fitting the statistical data, it shows that the probability distribution of time delay follows a gamma distribution which is related to the waiting time of Poisson distributed events. The fading frequency follows an exponential distribution, and the mean of multipath power attenuation decreases linearly with an increasing time delay. In addition, the detailed statistical characteristics for different elevations and orbits satellites is studied, and the parameters of each distribution are quite different. The research results give useful guidance for navigation simulator and receiver designers.

## 1. Introduction

The high-precision differential technique of global navigation satellite system (GNSS) has prompted the development of applications such as unmanned aerial vehicles (UAVs), autonomous land vehicle driving, and precise farming. The differential positioning technique can remove most of the errors, such as satellite ephemeris and clock, ionosphere, and troposphere, by using spatial or temporal correlations between the mobile receiver and the reference station. However, multipath is an exceptional interference because of its dependency on specific environments. Unfortunately, there is no method that can solve the multipath problem completely, especially in a severe multipath environment, such as an urban canyon area. Therefore, multipath is the last major error source that may prevent high accuracy in some circumstances.

An important multipath error elimination strategy is to design a suitable filtering according to multipath statistical features [1]. A similar example is the application of a mobile correspondence satellite, for which a land mobile channel model has been fully studied [2,3]. Research on the GNSS signal’s multipath statistical model is also necessary and important. However, there are a few reasons that make establishing the GNSS multipath models difficult. First, the GNSS signal is extremely weak when it arrives at the surface of the earth, which makes the reception and the analysis of the multipath more difficult. Second, the blend of the multipath signal and the line-of-sight (LOS) signal makes it more difficult to separate each component ray, especially when we are more interested in the near multipath, which lies within the one-chip extension. Third, the variety of application environments makes studying the multipath channel more difficult. Therefore, it is not easy to make significant progress in a short time.

Over the past several decades, researchers worldwide have made much progress in characterizing multipath signals. Normally, the study methods can be divided into two types. One type of method uses a ray tracing technique to precisely calculate all possible multipath signals for a specific environment. This type is very suitable for studying multipath characteristics under specific carriers, such as fighter and warship, since the 3-dimensional structure model of carriers can be built relatively precise [4]. The ray tracing technique is also proposed to study the code phase and carrier phase characteristics of multipath propagation, and the accuracy of the method is verified by real data [5,6]. Some algorithms are proposed to improve the positioning performance by using a 3D model [7]. However, it is very difficult to build a precise 3D model for a large-scale environment and use the ray tracing method, without even mentioning the many computations required. The practical applications of these approaches have many limitations, such as the fact that the surrounds need to be kept static. Thus, there is a need for new approaches to be proposed.

Another type of method uses signal estimations on sampled real multipath signals to directly study the characteristics of multipath parameters for interested environments. Since multipath code error is periodic for the static receiver, the error model is often used to mitigate the multipath error of pseudorange [8,9]. These research studies only consider the estimation error of the delay lock loop, which is caused by the static reflectors [10]. However, it is more important to analyze the detailed characteristics of a multipath caused by the environment, such as time delay, power attenuation, and fading frequency. To solve this problem, some theoretical models have been proposed to characterize near-surface reflectometry [11,12]. In addition, the German Aerospace Center (DLR) group emits impulse signals from a Zeppelin in different environments, and the impulse response data are sampled and recorded for analysis by multipath channel modelling [13]. According to these experimental data, some statistical models are built to characterize the echo number, delay, power and Doppler [14,15]. These statistical models are also studied in different environments and applications [16]. The achievements are included in the ITU-R report [17]. A satellite-to-indoor channel model is proposed for testing and validating range estimation algorithms [18]. To study the characteristics of the real signal, the GNSS group at Calgary University investigated the urban multipath channel characteristics, and their results showed the relationship among the code delay, fading frequency and carrier noise ratio [19]. The antenna is an important factor to consider in multipath interference, so a statistical model of multipath affection for antenna is proposed [20]. In conclusion, the statistical method is often used to study the multipath characteristics in a large-scale environment. However, the challenge of this method is to precisely obtain raw data about the multipath characteristics.

Although many achievements have been made in previous research, further research efforts in multipath channels are still required, especially for the urban environment. First, as new constellations are deployed, different orbit satellites, such as geosynchronous orbit (GEO), inclined geosynchronous satellite orbit (IGSO) and medium earth orbit (MEO), are put to work. There are not enough studies on the statistical multipath channels of different orbits, especially for the BeiDou Navigation Satellite System (BDS). Second, most researchers have focused on kinematic multipath channel models. Research on static multipath channel models is seldom seen in the literature. Third, the models developed by research groups such as DLR are based on data obtained from emulated signals that are emitted from a Zeppelin instead of a real GNSS signal. A research method based directly on real GNSS signals has not been widely used. Fourth, no empirical formulas have been proposed to describe the statistical characteristics of multipath parameters based on the experimental data. Targeting these goals, a signal sampling device is used to collect BDS B1I and GPS L1CA signals in a static urban canyon environment. Statistical models about multipath core parameters, including the time delay, the power attenuation and the fading frequency, are developed by analyzing and fitting those experimental data.

The paper is organized as follows. First, the tools that are used to extract raw multipath parameters from the real signal are introduced, and the performance is tested by a GNSS simulator. Next, the urban canyon environment, where the real multipath data are collected, is described, and the method used to process multipath raw data is illustrated. Then, the statistical analysis of multipath time delay, multipath power attenuation, and multipath fading frequency is described. Finally, some conclusions are summarized based on the experimental results.

## 2. Multipath Parameters Extraction Method

In the multipath environment, the received signal is composed of the LOS signal and multiple multipath rays. The compound signal can be expressed as [21]:

(1) $$s(t)=Ax(t-\tau_{0})\cos(\omega_{0}t+\theta_{0})+A\sum_{k=1}^{N}\alpha_{k}x(t-\tau_{0}-\tau_{k})\cos[\omega_{0}t+\theta_{0}+\Phi_{k}(t)]$$

where A and $\tau_{0}$ are the signal power and propagation time of LOS, respectively. The symbol *x*(.) represents the product of the navigation code and the spreading code. *N* is the multipath number. The parameter $\alpha_{k}$ is the amplitude attenuation proportionality coefficient. $\tau_{k}$ is the *k*-th multipath ray’s time delay relative to LOS, and $\Phi_{k}(t)$ is the *k*-th multipath ray’s carrier phase difference relative to LOS.

According to (1), it can be seen that the main parameters of multipath signal are $\tau_{k}$, $\alpha_{k}$ and $\Phi_{k}(t)$. Thus, in the following paper, time delay $\tau_{k}$, power attenuation $20\log\alpha_{k}$, and fading frequency $d\Phi_{k}(t)/2\pi dt$ are chosen as multipath parameter core features to be studied.

**Figure 1:**
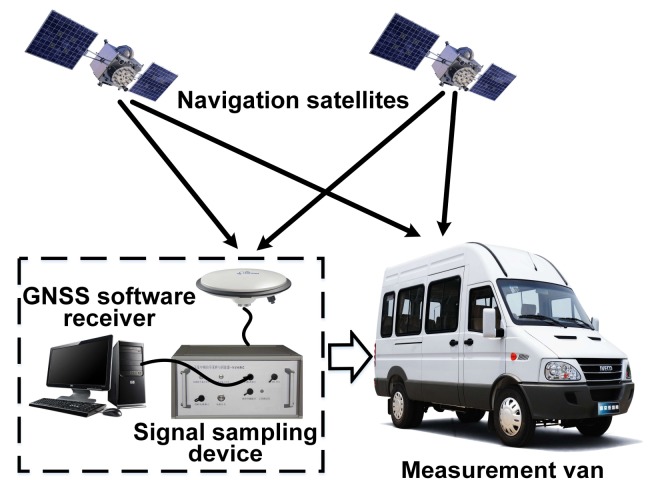
Global navigation satellite system (GNSS) signal intermediate frequency (IF) data recording platform.

To obtain multipath parameter features from the real satellite signal, a broadband intermediate frequency (IF) data sampling and recording device is designed to store both GPS L1CA and BDS B1I raw IF signals at the same time. A one-stage, the direct down-conversion in-phase/quadrature orthogonal sampling technique is adopted with a local oscillator (LO) frequency of 1568 MHz. The sampling frequency is 62 MHz complex samples per second, with 8-bit quantization resolution. The 1 TByte capacity is able to support more than 2 h of continuous signal recording. The specifications of the signal recording device are shown in Table 1. This device is mounted on a van to record data in the scenarios of interest, which is shown in Figure 1. A right-hand circular polarization (RHCP) antenna is mounted on the top of the van.

**Figure 2:**
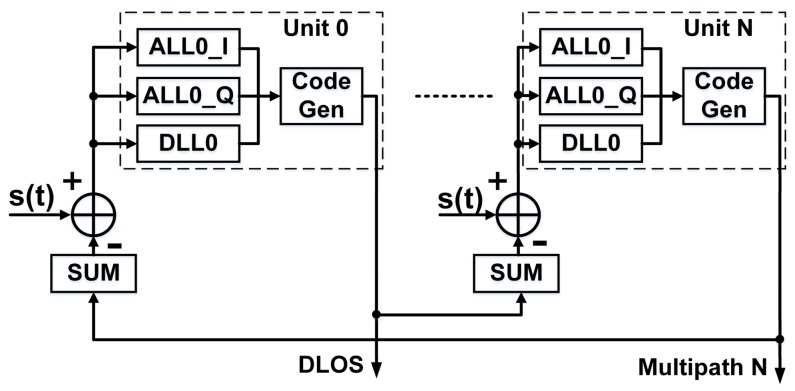
Block structure of Coupled Amplitude and Delay Lock Loop (CADLL).

After collection in the field, the IF data were exported and processed off-line with a software receiver developed by our group. One of the algorithms implemented in this receiver is the Coupled Amplitude and Delay Lock Loop (CADLL) multipath estimation method, which can estimate multipath parameters by processing the raw IF data [22,23]. The Block structure of the CADLL algorithm is shown in Figure 2. Instead of the traditional Delay Lock Loop (DLL), CADLL uses several units, which consist of one DLL and two Amplitude Lock Loops (ALL), to track the spreading code. Each unit can track one path ray, and unit always tracks the LOS signal. In every unit, DLL is used to estimate the code phase, and ALLs are used to estimate the normalized amplitude of I and Q branches. The time interval of each unit should be no less than 0.1 chip, so the maximum number *N* of multipath detection is ten in one chip. However, according to the analysis of experimental data, five channels for one satellite are enough to track most powerful multipath signals. Normally, the power of the rest multipath is too low to interfere with the positioning performance.

According to (1), the code phase delay of the *k*-th multipath ray, with respect to the LOS signal, is calculated as

(2) $$\tau_{k}={\hat{\tau }}_{k}-{\hat{\tau }}_{0},$$

where ${\hat{\tau }}_{0}$ and ${\hat{\tau }}_{k}$ are the estimated time delays of LOS and *k*-th multipath, respectively. The signal amplitude of each path ray is

(3) $$A_{k}=\sqrt{{\left({\hat{a}}_{k,I}\right)}^{2}+{\left({\hat{a}}_{k,Q}\right)}^{2}},$$

where ${\hat{a}}_{k,I}$ and ${\hat{a}}_{k,Q}$ are the estimated amplitudes of the I and Q branches of the *k*-th multipath, respectively. Then, the power attenuation proportionality coefficient is defined as

(4) $$Att_{dB}=20\log\alpha_{k}=20\log(A_{k}/A_{0}).$$

The carrier phase of each path ray is computed as

(5) $${\hat{\theta }}_{k}=\arctan2\left({\hat{a}}_{k,Q},{\hat{a}}_{k,I}\right).$$

Therefore, the multipath frequency fading of the *k*-th path ray is computed as

(6) $$f_{fading}^{k}=\frac{d\Phi_{k}(t)}{2\pi \cdot dt}=\frac{d\left({\hat{\theta }}_{k}(t)-{\hat{\theta }}_{0}(t)\right)}{2\pi \cdot dt}.$$

One hundred and twenty-seven correlators are used in CADLL to make the multipath-detection scope extend to 4 chips, which cover most of the possible multipath delays encountered in the urban environment. A Spirent GSS8000 simulator is used to test the performance of the CADLL algorithm. The configuration of the case is shown in Table 2.

**Figure 3:**
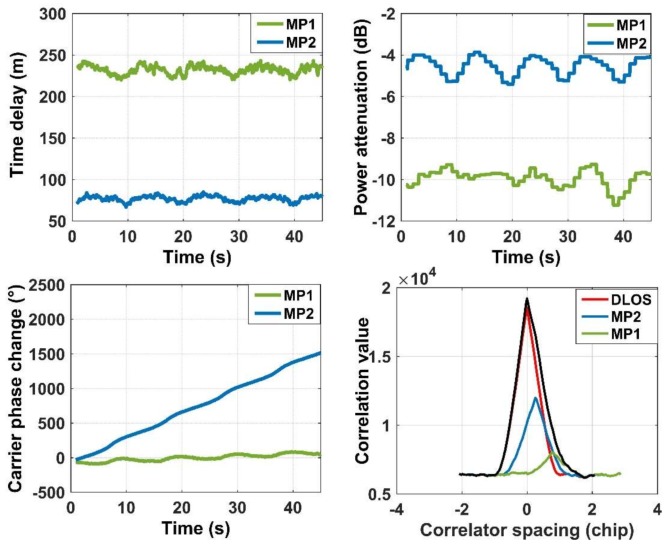
Time delay, power attenuation, carrier phase change and correlation shape of two multipath signals extracted by CADLL.

Figure 3 shows the estimation results of the simulated case processed by the CADLL algorithm. The subgraphs at the top-left, the top-right, the bottom-left and the bottom-right show the estimated multipath time delay $\tau_{k}$, the multipath power attenuation $Att_{dB}$, the multipath carrier phase difference $\Phi_{k}(t)$, and the correlation waveform, respectively. The carrier phase difference will be integrated if the multipath signal is continuous. The multipath fading frequency $d\Phi_{k}(t)/dt$ is the gradient of the carrier phase difference.

From Figure 3, it can be seen that the two multipath signals are correctly estimated. The green line represents multipath 1, and the blue line represents multipath 2. In the subgraph of the correlation waveform, the black curve, which represents the compound signal, is severely distorted. After the CADLL algorithm separated each path ray from the compound one, the correlation waveform of each path signal recovered to a regular triangular-wave. The average estimation errors for each multipath are listed in Table 3.

## 3. Description of the Research Campaign

**Figure 4:**
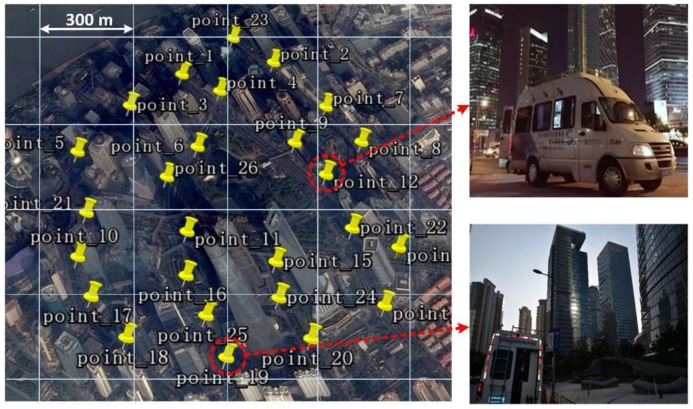
Satellite imagery of navigation signal collection spots and collecting live photos.

We chose Lujiazui, Shanghai, which is one of the most populated skyscraper areas in China, as the target urban canyon environment to study. This experiment area is located around 31°14′19.09′′ N, 121°29′58.77′′ E and covers approximately 1.7 square kilometers. We divided this area into 16 even cells, each of which covered approximately 0.1 square kilometers. On-field data collection occurred 26 times, all of which were marked on the Google map shown in Figure 4. Each cell area had an average of 1.6 spots of on-field data collection. For each on-field collection activity, more than 30 min of raw IF data for both GPS L1 and BDS B1 signals were recorded. All of these data were processed by the software receiver. More than 20,000 multipath signals were found from both constellations, which was large enough to build statistical models. The detailed number is shown in the Appendix A.

Among all the multipath detected, it was necessary to design a principle to separate different types of multipath signals before conducting a statistical analysis. We used two different sets of separation criteria for the GEO and non-geosynchronous orbit (NGEO) satellites because their motion status and multipath characteristics differed.

**Figure 5:**
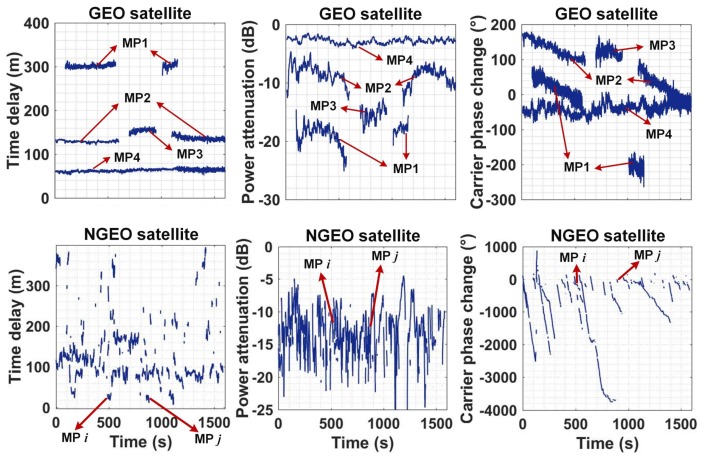
The multipath characteristics of one geosynchronous orbit (GEO) satellite and one non-geosynchronous orbit (NGEO) satellite at collection spot 1.

Figure 5 shows the raw multipath characteristic parameters of both GEO and NGEO signal. The top three panels are the GEO characteristics derived from the BDS PRN4 satellite, and the bottom three panels are the NGEO characteristics derived from the GPS PRN2 satellite. Referring to (1), each subplot gives a multipath delay $\tau_{k}$, power attenuation $20\log\alpha_{k}$, and carrier phase difference $\Phi_{k}(t)$ separately. The multipath fading frequency $d\Phi_{k}(t)/dt$ is the gradient of the carrier phase difference. It can be found that the characteristics of these two orbit satellites have significant differences.

The principle of separating a multipath signal refers to time delay, power attenuation, and fading frequency simultaneously. First, the multipath is segmented into different sections when any characteristic changed significantly, so many sections are obtained for the whole period. Then, some sections are combined and considered one multipath if the mean value of all three parameters is similar. The multipath characteristics of the NGEO satellite fluctuate more heavily than the GEO multipath, so the methods of separating the multipath are slightly different between GEO and NGEO satellites. The boundary parameters of segmenting and combining sections are shown as follows,

(1) The difference of code delay is larger than 0.1 chip;
(2) The difference of power attenuation is larger than 3 dB;
(3) The difference of multipath fading frequency is larger than 1 × 10^−4^ Hz (for GEO)/1 × 10^−2^ Hz (for NGEO).
(4) The interruption time is more than 1 min (only for NGEO).

In Figure 5, four multipath signals are detected for the GEO satellite, and both multipath 1 and multipath 2 contain two sections. The two sections are combined into one multipath because all of the characteristic parameters of the two sections are similar. However, for the NGEO satellite, multipath *i* and multipath *j* are considered two multipath signals, even though all of their feature parameters are similar.

In addition, if only non-line-of-sight (NLOS) signals can be received, this satellite signals will be excluded from the data set. For the NLOS signal, the pseudorange error is usually very large and the signal power is much lower. Thus, it can be easily detected by the signal power and residual value of pseudorange especially in the static case [24,25].

## 4. Statistical Model Analysis

We built some statistical models of multipath characteristics by analyzing the experimental data. The characteristics included multipath time delay, multipath power attenuation and multipath fading frequency. The validity of these models was proven by the chi-square test. The raw multipath data of all collection spots were mixed in the following analysis to achieve reasonable statistical models. According to the experiment results of all three satellite orbits, the statistical characteristics of time delay and power attenuation are the similar, while the characteristics of fading frequency have significant differences. Thus, the model comparison of three satellite orbits were analyzed only for the multipath fading frequency.

### 4.1. Multipath Time Delay

**Figure 6:**
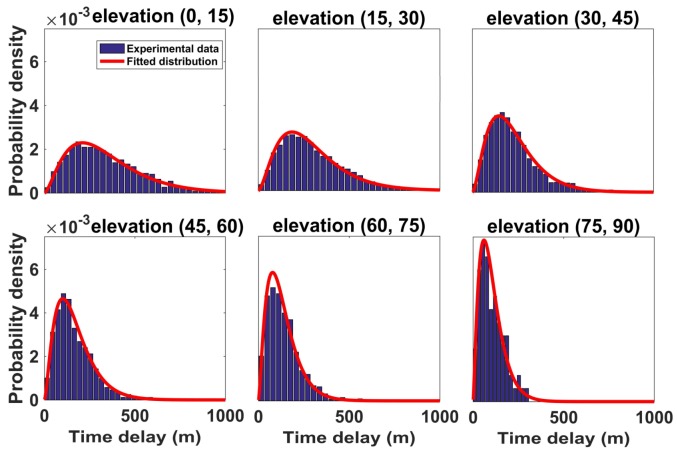
Statistical distribution of time delay in different elevation angle ranges.

We build the statistical distribution models for different elevation angle ranges because the multipath characteristics are closely related to the satellite elevation angle. Figure 6 shows the probability density distribution of multipath time delay with different satellite elevations.

In Figure 6, the histogram is obtained from statistical experimental data, and the Gamma distribution is adopted to fit the data. The probability density function of the Gamma distribution is as follows:

(7) $$\begin{array}{cc} f(x;\gamma ,\varsigma )=\frac{1}{\Gamma (\gamma )\cdot \varsigma^{\gamma }}\cdot x^{\gamma -1}\cdot e^{-\frac{x}{\varsigma }} & x\in (0,\infty ) \end{array}$$

where *x* is the time delay. Γ(·) is the Gamma function. γ and ς are the parameters which is different for different elevation angles. The multipath time delay is shorter when the satellite elevation is higher. The distribution parameters under different elevations are given in Table 4. The table shows that the shape parameters γ are similar under different elevation angles but that the scale parameters ς are inversely proportional to the elevation.

To verify that the fitted distribution is acceptable, the chi-square test is used in every probability distribution model [26]. The formula of the chi-square test is as follows:

(8) $$\chi^{2}=\sum_{i=1}^{k}\frac{{\left(f_{i}-n\cdot p_{i}\right)}^{2}}{n\cdot p_{i}},$$

where $f_{i}$ is the sample number of the *i*th interval and $p_{i}$ is the theoretical probability of *i*th interval. *n* is the sample size, and *k* is the number of intervals. The result should follow a chi-square distribution with k−1 degrees of freedom.

Since this paper mainly focus on the empirical model based on the experiment measurements, a common method of studying distribution function is to use all potential functions with similar trend to fit the statistical histogram. The chi-square test result is used to represent the fitting consistency of each function. It can be found that the overall probability distribution of time delay first rise and then drop, so the following six distribution functions are chosen to fit the data. The test result of time delay distribution fitted by these functions are shown in Table 5. According to the value of Table 5, it can be seen that the Gamma distribution is the most suitable for the time delay. In addition, the gamma distribution is equal to the sum of multiple independent and identically distributed exponential distribution, and the exponential distribution is commonly used to describe the waiting time of Poisson distributed events. Thus, the Gamma distribution Gamma(γ,ς) can represent the waiting time of γ-th event. To interpret the gamma distribution of multipath time delay from a physical standpoint, it can be suggested that the multipath signal may be reflected γ times in statistical analysis. Table 4 indicates that the statistical reflecting times are between 2 and 3 times for different elevations.

The chi-square test of time delay with different elevation fitted by the Gamma distribution are shown in Table 6. It shows that most of the test results are less than $\chi_{0.01}^{2}(k-1)$, which confirmed that each distribution function is credible. In addition, it is difficult to calculate the chi-square test when the raw experimental data are insufficient. However, the overall trend of the histogram is consistent with each distribution.

**Figure 7:**
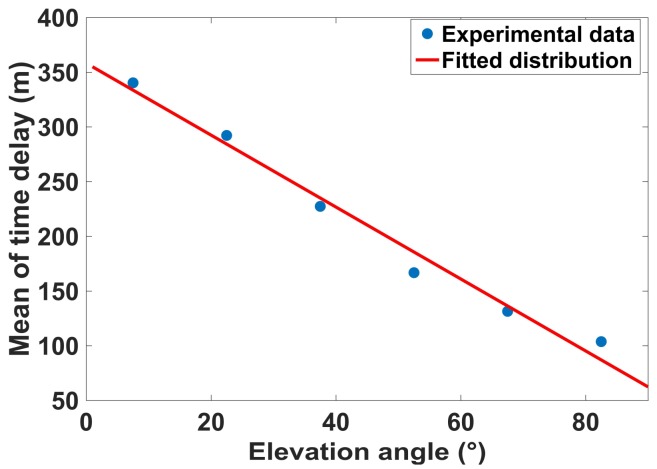
The fitted distribution for mean of multipath time delay with different elevation angles.

Figure 7 shows the mean of multipath time delay with different elevation angles. The mean value is nearly linearly decreasing with elevation angle increases. The fitted distribution is given as follows:

(9) f(x;a,b)=a·x+b

where *x* is the elevation angle. The parameters a=−3.3 (−3.9, −2.7) and b=358.3 (325.3, 391.3), with 95% confidence bounds. This illustrate that the mean of multipath time delay decreases approximately 3 m when the satellite elevation increases 1 degree. The mean of multipath time delay for all elevation angles is approximately 200 m.

**Figure 8:**
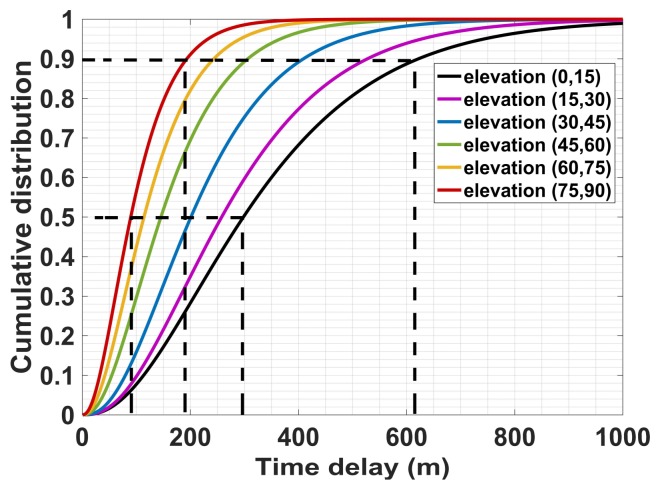
Cumulative distribution of time delay with different elevation angles.

Figure 8 gives the cumulative distribution probability (CDF) of multipath time delay with different elevation angles. It illustrates that the cumulative probability is larger as the satellite elevation angle increases. The multipath time delays corresponding to 50% and 90% cumulative probability are marked in this figure. More than half of the delay is less than three hundred meters, and most of the delay is less than six hundred meters.

### 4.2. Multipath Power Attenuation

**Figure 9:**
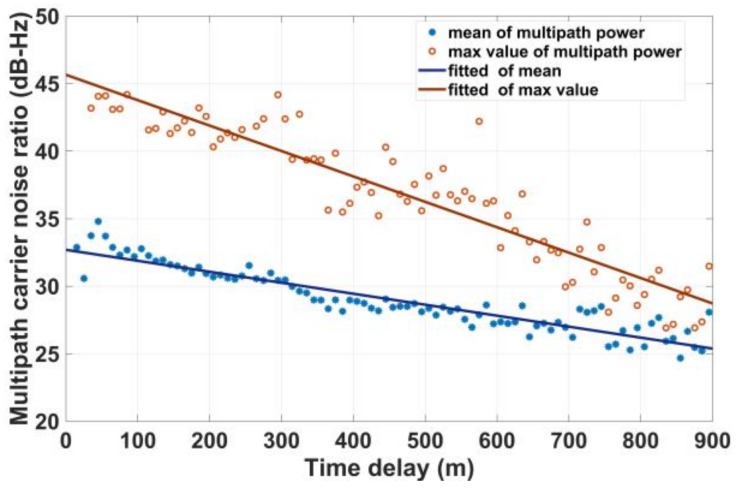
Maximum and mean of multipath power as a function of time delay.

In this section, the multipath power attenuation will be studied. Figure 9 gives the mean and maximum of multipath power with different multipath time delays. The mean and maximum of multipath power are a linearly decreasing function of time delay. There are two reasons for this phenomenon. One reason is that the power of the electromagnetic wave propagation attenuates in the atmosphere. The other important reason is that the multipath signal is shadowed with a higher probability when the multipath delay is longer. The fitted curve is expressed as follows:

(10) f(x;a,b)=a·x+b

where *x* is the time delay, and the parameters *a* and *b* are given in Table 6. The table indicates that most of the multipath power within one chip delay is more than 30 dB and that the maximum could be more than 40 dB. It is also observed that the multipath power is always less than 45 dB but that the LOS signal power may exceed 50 dB.

**Figure 10:**
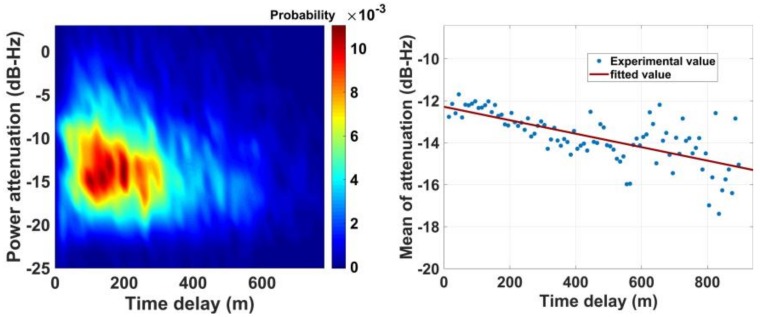
Probability of multipath power attenuation as a function of time delay.

Figure 10 gives the probability distribution of multipath power attenuation. According to the left panel, the attenuation is mainly distributed between −10 dB and −17 dB, and the multipath power may occasionally be higher than LOS. The right panel gives the mean distribution of power attenuation as a function of time delay, and it is a linearly decreasing function of time delay.

All of the fitted parameters are shown in Table 7.

During our experiments, the statistical characteristics of time delay and power attenuation are not distinct for different orbit satellites. This is reasonable because the time delay and power attenuation are largely related to the distribution and materials of surrounding reflecting/scattering objects other than the satellite orbits. However, when the antenna is static, the multipath fading frequency mainly depend on the satellite velocity relative to earth. Since the satellite velocities to earth of GEO, MEO, and IGSO are different, the different fading frequency behaviors for different orbits are discussed.

### 4.3. Multipath Fading Frequency

Multipath fading frequency affects the periodic behavior of observation errors caused by multipath. There are three major factors that influence multipath fading frequency: the position and velocity of the satellite, the position and velocity of the receiver antenna, and the reflector position. In this paper, the statistical multipath fading frequency characteristics for MEO, IGSO and GEO orbits are discussed.

**Figure 11:**
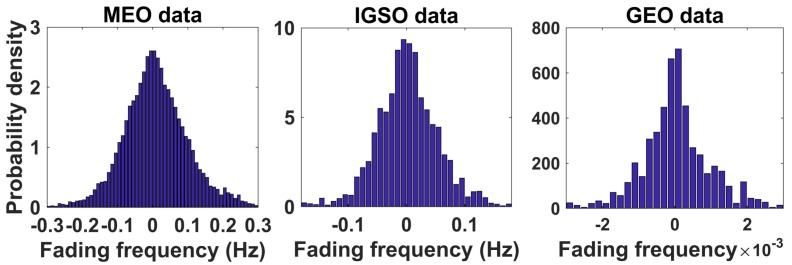
Histograms of multipath fading frequency distribution for three orbits.

Figure 11 gives the fading frequency distributions obtained from experimental data for different orbits in the form of histograms. It shows that the multipath fading frequency is axisymmetric approximately 0, so we take the absolute value of the multipath fading frequency for convenience in the following. The probability density is a monotonic decreasing function.

**Figure 12:**
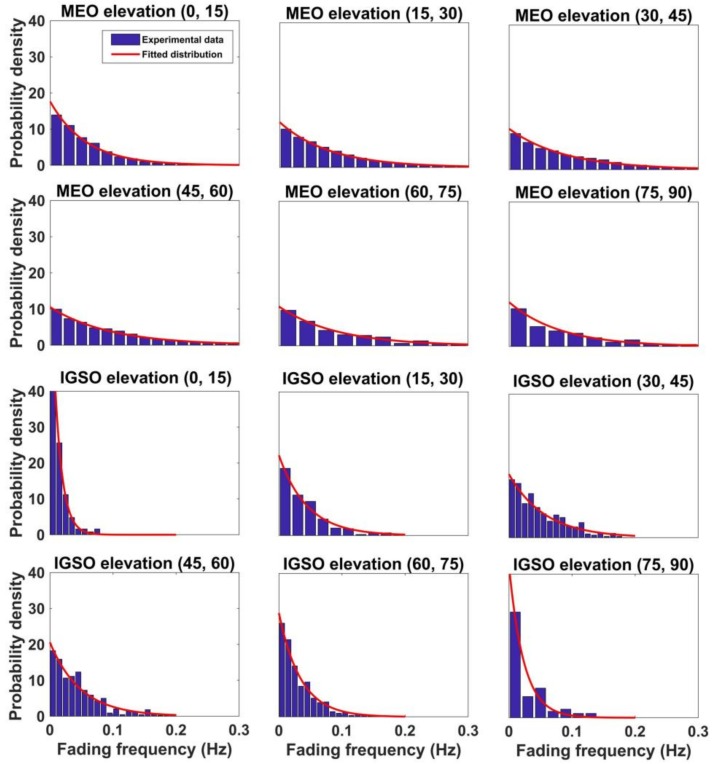
Statistical probability distribution of multipath fading frequency for the medium earth orbit (MEO) and inclined geosynchronous satellite orbit (IGSO) satellite in different elevations.

Figure 12 shows the probability distribution of the multipath fading frequency of the MEO and IGSO satellite at different elevations. The exponential distribution is used to fit the experimental data. The statistical experimental data of IGSO are not very smooth, as the MEO data are, because there are only five IGSO satellites. Thus, the number of multipath detected from the IGSO satellite is smaller than that of MEO. Although the raw experimental data of the IGSO satellite are insufficient to obtain a smooth distribution, the conclusions are still reasonable.

Considering the probability of the multipath fading frequency with a positive sign and negative sign is the same, the fitted probability distribution function is given as follows:

(11) $$f(x;\lambda )=\frac{1}{2}\lambda \cdot e^{-\lambda \cdot \left|x\right|}$$

where *x* is the fading frequency. The parameter λ for different elevation angles is given in Table 7. Most of the values of the NGEO multipath fading frequency are smaller than 0.3 Hz, which shows that the multipath fading frequency is small for a static receiver. The mean of each distribution can be calculated by 1/λ, and it can be concluded that mean period of NGEO multipath variation is less than one minute.

**Figure 13:**
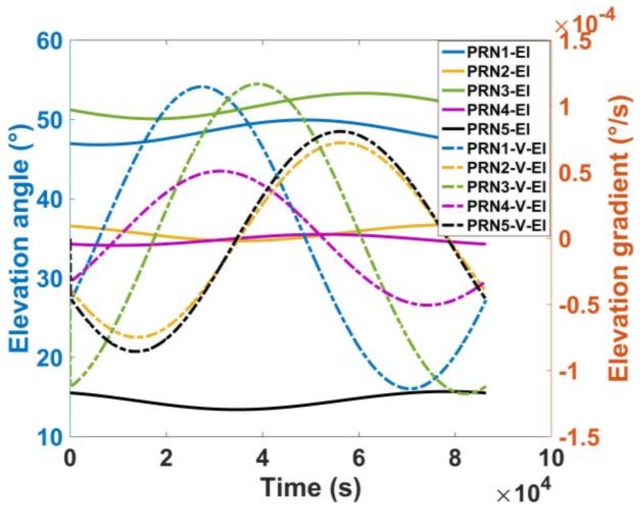
Elevation and variation velocity of the five BeiDou GEO satellites in one day.

The GEO satellite is almost static with respect to earth, and its elevation angle and azimuth angle change slowly. There are five GEO satellites in the BDS system, and the variation range of their elevation angle is less than 4 degrees. Figure 13 gives the elevation angle and variation velocity of elevation for five GEO satellites in one day.

**Figure 14:**
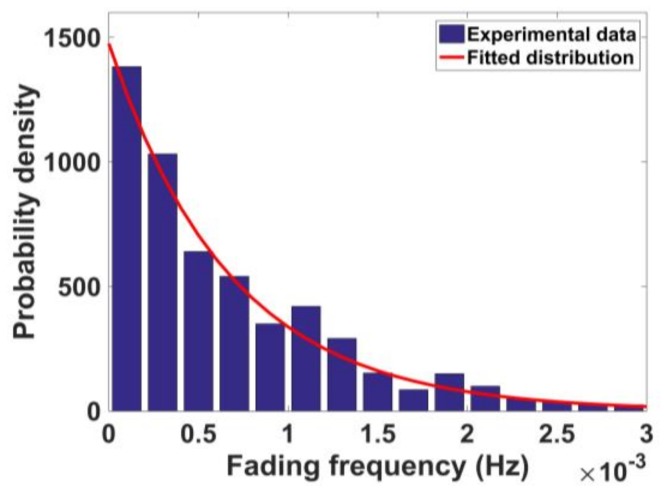
Statistical probability distribution of multipath fading frequency for the GEO satellite.

The experimental statistical distribution of the multipath fading frequency for GEO satellite is given in Figure 14. However, it is difficult to analyze the relationship between the multipath fading frequency and elevation angle because the variation of the GEO elevation angle is very small. The exponential distribution is also used to match the experimental data of multipath fading frequency.

The chi-square test is also used to test the goodness of fit in the fading frequency distribution in Table 8. The result shows that the parameters values matched the experimental data very well.

**Figure 15:**
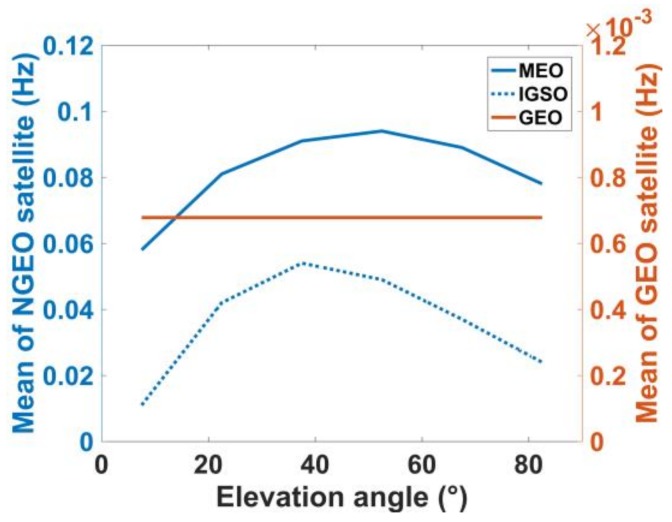
Mean of multipath fading frequency for different satellite orbits.

Figure 15 shows the mean multipath fading frequencies for three orbit types under different elevation angles. The blue solid line, blue dashed line and red solid line denote the mean fading frequency of the MEO, IGSO and GEO satellites, respectively. The mean fading frequency of the MEO multipath is the maximum. The mean fading frequency of the IGSO satellite is approximately half of that of MEO. In addition, the mean fading frequency of the GEO satellite is approximately one percent of that of NGEO (MEO and GEO). This implies that the variation period of observation error of the GEO satellite may last tens of minutes in most cases.

### 4.4. Model Comparison

**Figure 16:**
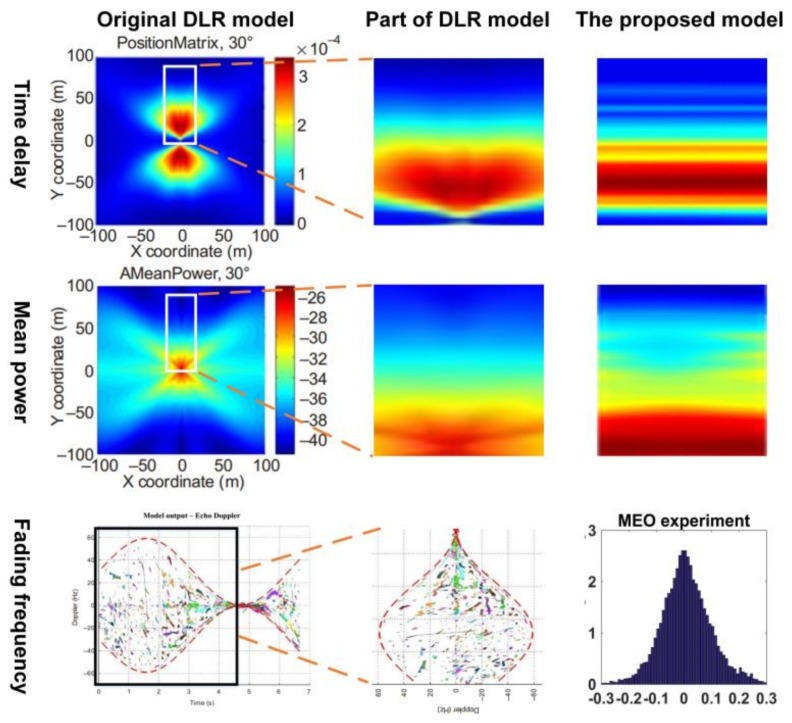
Comparison between our models and the German Aerospace Center (DLR) models for the characteristics of time delay, mean power and fading frequency.

To give deeper insight into the statistical models developed in this paper, we compared our models with the models developed by the DLR group. The DLR group studied multipath models in several types of carriers and environments, and we chose the one obtained from the urban environment for comparison. The left three plots of Figure 16 show the distribution of horizontal reflector coordinates, mean of power and echo Doppler of the DLR models cited from ITU-R P.2145-1 [13], and the right three plots are our corresponding distributions. It has to be mentioned that the reflector horizontal coordinates distribution instead of the multipath delay distribution is obtained in DLR model because of different experiment instruments used in their research. The *X* and *Y* coordinates are the horizontal position of reflection points with respect to the receiver. Although longer distance from reflector to receiver does not necessarily produce larger multipath delay, it has large possibility that further reflector corresponds to large delay. Thus, it is reasonable to compare the overall trend of these two distributions. To compare the models in only one dimension for the time delay and mean power, we choose the DLR model with the coordinate interval (x=0, y>0) which are shown in the white rectangular box. The middle three plots are the rectangular part of origin models which are zoomed in.

We present the delay distribution and the power attenuation distribution from our research in a thermodynamic chart (shown in the right three plots of Figure 16) to compare the two sets of models in the same perspective. Since both the delay and the power attenuation have a one-dimensional distribution in our model, only the *Y* axis has physical meanings that corresponded to the delays, and the *X* axis do not contain any information about the distribution. The distributions are supposed to form a line in the two-dimensional thermodynamic charts. In order to make thermodynamic charts more readable, we use strips to replace the supposed lines.

According to the time delay models (top three plots), the possibility of very short delays (or very near reflectors) from the antenna is quite low (dark blue shown in the charts). Then, the possibility increases as the delay increases (or the distance of reflector increases), but after a certain peak, the possibility decreases as the delay increased further. It can be seen that this trend occurs to the models of both the DLR and this paper. When we look at the power attenuation distribution (middle three plots), it can be found that both the models of the DLR and this paper follows decreasing trend as the delay increase. For fading frequency model (bottom three plots), since the DLR models are developed based on a moving carrier but the models in this paper focused on the static platform, the Doppler (or the multipath fading) distributions cannot be compared. However, we still determine that both models regarding the multipath Doppler are symmetrical around the zero axis.

Overall, although the raw data are collected in different types of environment and different cities, the multipath delay and power attenuation derived in this paper are consistent with the distribution trend found by other research groups. Thus, in other types of environment, it can be believed that the distribution functions are also suitable for the multipath characteristics, but different parameters are needed.

## 5. Conclusions

We mainly studied the statistical distribution models of multipath characteristics based on the experimental data in an urban canyon environment. The characteristics contained the multipath time delay, multipath power attenuation, and multipath fading frequency. In addition, the relationship between the statistical distribution and elevation or orbits was analyzed. Several main conclusions can be drawn:

- The probability distribution of multipath time delay followed a gamma distribution, and the mean time delay is inversely proportional to the elevation angle.
- The mean multipath power attenuation linearly decreases as the multipath time delay increases, and the multipath attenuation is normally less than −17 dB.
- The probability distribution of the multipath fading frequency is axisymmetric around 0 Hz, and it follows a double-sided exponential distribution.
- For different elevations and different satellites orbits, the type of distribution function to characterize each core parameters is the same, but the feature parameters are different.

The contribution of this work provides some complements to the existing multipath models. The novel probability functions of multipath time delay and fading frequency are proposed. The results can be used in some research fields, such as multipath simulator, multipath mitigation, and spoofing detection. For example, the statistical model can help us to design the initial parameters for algorithms based on filter theory or estimation theory. The distribution characteristics can also be used to distinguish between a spoofing signal and multipath. In the future, we will continue this research in different types of environments.

## Figures and Tables

**Table 1:** Parameters of the signal recording device.

Signal Sampling Device		GNSS Antenna (NovAtel GPS-703-GGG)	
Constellation	BDS B1 & GPS L1	Polarization	RHCP
Sampling frequency	62 MHz	Peak gain	5.0 dBi
Signal bandwidth	20 MHz	Directivity	Horizontal omnidirectional
Bit width	8 bit	Axial ratio	<2.0 dB

**Table 2:** Configuration of Spirent GSS8000 simulator.

Constellation	GPS L1CA/PRN2	
LOS CNR	38 dB	
Multipath number	MP 1	MP 2
Multipath delay	0.8 chip/234 m	0.3 chip/87 m
Power attenuation	−10 dB	−5 dB
Fading frequency	0.01 Hz	0.1 Hz

**Table 3:** Estimation errors of the CADLL algorithm.

Multipath		Delay	Attenuation	Frequency
Multipath 1	Estimation	232 m	−10.2 dB	0.00995 Hz
	Error	2 m	0.2 dB	5 × 10^−5^ Hz
Multipath 2	Estimation	81 m	−4.5 dB	0.09978 Hz
	Error	6 m	0.5 dB	2.3 × 10^−4^ Hz
Performance		<10 m	<1.5 dB	<1%

**Table 4:** Distribution parameters of time delay with different elevations.

Elevation	γ	ς
(0, 15)	2.62	129.83
(15, 30)	2.77	105.52
(30, 45)	2.81	80.93
(45, 60)	2.56	65.12
(60, 75)	2.47	53.22
(75, 90)	2.40	43.24

**Table 5:** Chi-square test result of time delay distribution fitted by six different functions.

Function	Gamma	Burr	Rayleigh	Rice	Log-Logistic	Weibull
Result	34.2	61.6	362.2	361.8	106.1	100.9

**Table 6:** Chi-square test result of distribution with different elevations.

**Elevation**	**(0, 15)**	**(15, 30)**	**(30, 45)**
$\chi^{2}/\chi_{0.01}^{2}/k-1$	31.6/46.9/27	28.1/46.9/27	34.7/46.9/27
**Elevation**	**(45, 60)**	**(60, 75)**	**(75, 90)**
$\chi^{2}/\chi_{0.01}^{2}/k-1$	32.2/42.9/24	37.0/41.6/23	-

**Table 7:** Fitted parameters of multipath power.

Parameters	*a* (95% Confidence)	*b* (95% Confidence)
Mean of multipath power	−0.0081 (−0.0088, −0.0074)	32.7 (32.3, 33.1)
Maximum of multipath power	−0.018 (−0.020, −0.016)	45.7 (44.5, 46.5)
Mean of power attenuation	−0.0032 (−0.0039, −0.0025)	−12.3 (−12.7, −11.9)

**Table 8:** Distribution parameter of multipath fading frequency.

Elevation Range	(0, 15)	(15, 30)	(30, 45)	(45, 60)	(60, 75)	(75, 90)
MEO/λ	17.2	12.3	10.9	10.6	11.2	12.8
IGSO/λ	90.9	23.8	18.5	20.4	27.0	41.7
GEO/λ	1495.9					

## Appendix A

**Table A1:** Statistics of detected multipath echoes.

Place/Type	Echo Number	Place/Type	Echo Number	Place/Type	Echo Number
Spot 1	701	Spot 10	1114	Spot 19	610
Spot 2	661	Spot 11	2114	Spot 20	537
Spot 3	1091	Spot 12	1643	Spot 21	756
Spot 4	700	Spot 13	389	Spot 22	922
Spot 5	821	Spot 14	599	Spot 23	465
Spot 6	786	Spot 15	624	Spot 24	389
Spot 7	1061	Spot 16	307	Spot 25	596
Spot 8	1581	Spot 17	962	Spot 26	1099
Spot 9	1013	Spot 18	929		
GEO Sat.	513	IGSO Sat.	2622	MEO Sat.	19,335
BDS Constellation		8389	GPS Constellation		14,081
Total number			22,470		
